# Comparison and Correlation of the Donor–Recipient Interface Changes and Visual Outcomes Between nDSEK and DSEK

**DOI:** 10.1155/joph/2066562

**Published:** 2025-03-12

**Authors:** Minghai Huang, Thuthuy Hoang, Guina Yin, Yanqing Liang, Zhifeng Wu, Jian Teng, Zhuoyuan Zhang, Dongmei Wei

**Affiliations:** Nanning Aier Eye Hospital, Nanning 530015, China

**Keywords:** corneal endothelial decompensation, Descemet stripping endothelial keratoplasty, in vivo laser confocal microscopy, non-Descemet stripping endothelial keratoplasty

## Abstract

**Purpose:** This study aimed to compare the donor–recipient interface changes between non-Descemet stripping endothelial keratoplasty (nDSEK) and Descemet stripping endothelial keratoplasty (DSEK) and assess their correlation with the postoperative best spectacle-corrected visual acuity (BCVA).

**Methods:** This retrospective study collected clinical data on patients with corneal endothelial decompensation who underwent either nDSEK or DSEK between August 2019 and April 2023. The donor–recipient interface particle density, interface haze, visual outcome, and graft dislocation were compared between nDSEK and DSEK groups.

**Results:** A total of 66 eyes from 66 patients (nDSEK *n*: 31 eyes and DSEK *n*: 35 eyes) were included. At 12 months postoperatively, nDSEK had a mean interface particle density of 631.97 ± 143.95 particles/mm^2^, significantly higher than DSEK's 518.20 ± 121.72 particles/mm^2^ (*p*=0.001). The interface haze was also greater in nDSEK (78.16 ± 13.74) compared with DSEK (64.21 ± 14.78) (*p* < 0.001). BCVA improved similarly in both groups, with nDSEK changing from 1.92 ± 0.26 to 0.37 ± 0.11 and DSEK from 1.85 ± 0.24 to 0.34 ± 0.10 (*p*=0.149). Correlation analysis revealed a significant association between interface particle density and interface haze in both the nDSEK (correlation coefficient: 0.716, *p* < 0.001) and DSEK (correlation coefficient: 0.618, *p* < 0.001) groups. However, there was no significant correlation between interface particle density and postoperative BCVA for either the nDSEK (correlation coefficient: −0.028, *p*=0.883) or DSEK (correlation coefficient: 0.111, *p*=0.525) group. Similarly, no significant correlation was found between interface haze and postoperative BCVA in both groups (nDSEK: correlation coefficient: −0.080, *p*=0.670 and DSEK: correlation coefficient: −0.210, *p*=0.227). Graft dislocation rates were comparable: 3.2% in nDSEK and 2.9% in DSEK (*p*=0.931).

**Conclusion:** nDSEK exhibited more interface particles and haze than standard DSEK, but visual outcomes and graft attachment were similarly effective.

## 1. Introduction

Endothelial keratoplasty has become the preferred surgical approach for managing corneal endothelial decompensation. Over time, surgical techniques have evolved from early methods like deep lamellar endothelial keratoplasty (DLEK) and posterior lamellar keratoplasty (PLK), which involved removing the recipient's posterior stromal discs, Descemet membrane, and endothelial cells complex. These techniques have been superseded by Descemet stripping endothelial keratoplasty (DSEK), Descemet stripping automated endothelial keratoplasty (DSAEK), and Descemet membrane endothelial keratoplasty (DMEK). Unlike the earlier methods, these newer approaches typically involve removing only the recipient's Descemet membrane and endothelial cells complex, as leaving these intact at the donor–recipient interface was thought to hinder graft adhesion and could lead to graft detachment [1]. However, contrary to earlier assumptions, histologic findings of Caldwell et al. in DSEK suggest that retaining the recipient's Descemet membrane may not necessarily impede graft adhesion, thereby raising the possibility that removal of Descemet membrane may be unnecessary for endothelial transplantation [2]. Several studies [3–5] have demonstrated comparable outcomes between modified techniques that omit these steps and standard DSEK or DSAEK procedures, such as non-Descemet stripping endothelial keratoplasty (nDSEK) and non-Descemet stripping automated endothelial keratoplasty (nDSAEK). These modified techniques may be suitable when the recipient's Descemet membrane shows no significant abnormalities. However, there remains ongoing controversy regarding the retention of the recipient's Descemet membrane and endothelial cell complex during endothelial keratoplasty. Comparative morphological studies between these modified techniques and standard procedures for treating corneal endothelial decompensation are still quite limited.

Therefore, our study aimed to retrospectively compare the morphological changes at the donor–recipient interface using in vivo laser confocal microscopy between groups undergoing nDSEK and those undergoing standard DSEK for the treatment of corneal endothelial decompensation. In addition, we sought to correlate these findings with postoperative best spectacle-corrected visual acuity (BCVA).

## 2. Materials and Methods

The data for this retrospective comparative cohort study were retrieved from the records of the Nanning Aier Eye Hospital in China. The subjects were individuals with corneal endothelial decompensation who underwent either nDSEK or standard DSEK procedures between August 2019 and April 2023 at the same hospital. The study received approval from the Medical Ethics Committee of Nanning Aier Eye Hospital, adhered to the principles of the Declaration of Helsinki, and informed consent was obtained from all the patients. Inclusion criteria encompassed individuals older than 18 years, eyes with corneal endothelial decompensation treated with either nDSEK or standard DSEK. Exclusion criteria included eyes with uncontrolled glaucoma, amblyopia, significant macular degeneration, optic neuropathy, subepithelial fibrosis or corneal scarring on the optic axis, Fuchs' endothelial dystrophy, significant Descemet membrane abnormalities, retrocorneal fibrous membrane, history of prior penetrating keratoplasty, cases of primary and secondary graft failure occurring within the first 12 months postoperatively, or inability to complete a minimum 12-month follow-up. All surgical procedures were performed by Dr. Minghai Huang at Nanning Aier Eye Hospital. Data on demographics, visual acuity, donor–recipient interface changes, and graft dislocation were extracted from medical records.

### 2.1. Preparation of Donor Endothelial Graft

The donor corneas used in this study were allocated by the Nanning Aier Eye Bank from voluntary donors, and all donors or their next of kin provided written informed consent that was freely given.

The endothelial graft was prepared manually in accordance with the technique described by Price and Price [6]. To summarize, a peripheral groove was made in the cornea using a knife marked with a 350 μm scale, followed by separation of the corneal lamella using a curved lamellar dissector. After achieving complete separation, the endothelial graft was excised from the posterior corneal surface using an 8.0 mm trephine (Barron vacuum donor cornea punch) and utilized for either nDSEK or standard DSEK procedures.

### 2.2. Surgical Procedure

In standard DSEK, a 4.0 mm superior scleral tunnel incision was created, and with the assistance of cohesive viscoelastic agents, the recipient's Descemet membrane and endothelium were initially stripped from the central cornea using a reverse Sinsky hook, followed by removal from the anterior chamber. A peripheral iridectomy was performed at six o'clock to prevent pupillary obstruction. An anchoring 10-0 nylon suture stitch was placed on the edge of endothelial graft. The endothelial graft on the Busin glide was then implanted into anterior chamber using a suture pull-through technique, as previously described by Hong et al. [7]. Finally, the endothelial graft was attached to the recipient cornea by fixing it with sterile air, which almost completely filled the anterior chamber. In contrast, nDSEK did not involve stripping and removing the recipient's Descemet membrane and endothelium complex. Consequently, the subsequent steps were identical to those of standard DSEK. After surgery, patients were instructed to maintain a supine position for 2 days.

During follow-up, patients underwent assessments including uncorrected visual acuity (UCVA) and BCVA, along with examinations using a slit-lamp (Topcon Corporation, Tokyo, Japan), anterior segment optical coherence tomography (AS-OCT, Heidelberg Engineering GmbH, Germany), and in vivo laser confocal microscopy with the Heidelberg Retina Tomograph 2 Rostock Cornea Module (HRT2-RCM, Heidelberg Engineering GmbH, Germany). For statistical analysis, decimal visual acuity was converted to the logarithm of minimal angle of resolution (logMAR). Considering the potential impact of graft dislocation on interface quality, cases of graft dislocation that occurred postoperatively were excluded from the statistical analysis of interface haze and particle density. Measurements taken at the 12-month postoperative follow-up were compared and analyzed between the nDSEK and standard DSEK groups.

The central corneas of subjects undergoing nDSEK or standard DSEK were examined for specific corneal layers using the HRT2-RCM. This device utilizes a 60× water-immersion objective (Olympus Europa GmbH, Hamburg, Germany) and a 670-nm diode laser as a light source, offering a viewing area of 400 × 400 μm. Images of the donor–recipient interface were processed and analyzed by two investigators, who were blinded to the patient group, conducting the image analysis retrospectively using ImageJ software, available at https://rsb.info.nih.gov/ij (National Institutes of Health, Bethesda, MD, USA). Quantitative analysis included measuring the intensity of donor–recipient interface haze in pixel intensity units, rated on a scale from 0 (pure black) to 255 (pure white). In addition, donor–recipient interface particle density was calculated based on particle counts using ImageJ. Statistical analyses were conducted using IBM SPSS Statistics (Version 22, IBM Corp., Armonk, NY, USA). Continuous values were expressed as the mean ± standard deviation. Demographic characteristics and baseline clinical data were analyzed, with categorical data such as the dislocation status, gender proportions, and lens status evaluated using Chi-square tests. The normality of continuous data, including the age of the corneal donors, endothelial cell density (ECD) of donors, interface haze, interface particle density, and converted visual acuity (LogMAR), was assessed through PP plots, QQ plots, and the Kolmogorov–Smirnov test, which indicated that these data approximated a normal distribution. Independent sample *t*-tests were conducted to analyze age, donor age, donor ECD, interface haze, and interface particle density. The visual outcomes in UCVA and BCVA over time in the two groups were compared using repeated measures analysis of variance (ANOVA). In addition, Pearson correlation analysis was used to assess the relationship between interface particle density, donor–recipient interface haze, and BCVA. A *p* value less than 0.05 was considered statistically significant.

## 3. Results

### 3.1. Demographic Characteristics and Baseline Clinical Data of Patients

During the study period, our hospital performed corneal endothelial transplantation on 96 patients (96 eyes) with corneal endothelial decompensation. According to the inclusion criteria, 94 patients were screened (age < 18 years,*n* = 2). A total of 28 patients were excluded (uncontrolled glaucoma, *n* = 1; macular degeneration, *n* = 2; optic neuropathy, *n* = 1; subepithelial fibrosis or corneal scarring on the optic axis, *n* = 2, Fuchs' endothelial dystrophy, *n* = 4; history of prior penetrating keratoplasty, *n* = 1; graft failure, *n* = 4; and < 12 months of follow-up, *n* = 13). The study finally included a total of 66 eyes from 66 patients (nDSEK: 31 eyes and DSEK: 35 eyes) according to the inclusion criteria and exclusion criteria. The mean age of patients was 61.97 ± 17.15 years in the nDSEK group (range: 28–93 years) and 61.51 ± 13.59 years in the standard DSEK group (range: 32–84 years), with females comprising 35.5% in the nDSEK group and 54.3% in the DSEK group. The mean donor age was 45.61 ± 11.91 years (range: 19–61 years) for the nDSEK group and 46.51 ± 9.55 years (range: 28–65 years) for the DSEK group. The mean donor ECD was 2816.77 ± 142.59 cells/mm^2^ for the nDSEK group and 2861.06 ± 143.70 cells/mm^2^ for the DSEK group. Preoperative BCVA and lens status are shown in Table 1. There were no statistically significant differences between the nDSEK and DSEK groups in terms of age (*p* = 0.905), sex (*p* = 0.126), donor age (*p* = 0.735), donor ECD (*p* = 0.214), preoperative BCVA (*p* = 0.218), and preoperative lens status (*p* = 0.591) (Table 1).

nDSEK: non-Descemet stripping endothelial keratoplasty; DSEK: Descemet stripping endothelial keratoplasty; ECD: endothelial cell density; BCVA: best corrected visual acuity; LogMAR: logarithm of minimal angle of resolution; age and BCVA are expressed as mean ± standard deviation, gender and lens status are presented as counts and percentages.

### 3.2. Clinical Assessments Involved General Observations Conducted via Slit-Lamp Microscopy, AS-OCT, and In Vivo Laser Confocal Microscopy Using the HRT2-RCM

Upon examination using slit-lamp microscopy and AS-OCT at the 12-month follow-up, all cases demonstrated corneal transparency with secure adhesion of the endothelial graft to the recipient bed. Under slit-lamp microscopy, no clinically visible differences were observed between the nDSEK and DSEK groups regarding corneal transparency and the donor–recipient interface. In the AS-OCT assessment, we observed a homogeneous band of moderate to low reflectivity at the donor–recipient interface in the nDSEK eyes, which is presumed to represent the recipient's Descemet membrane. In contrast, no similar band was detected in the DSEK eyes (Supporting Figure S2).

**Figure 1:**
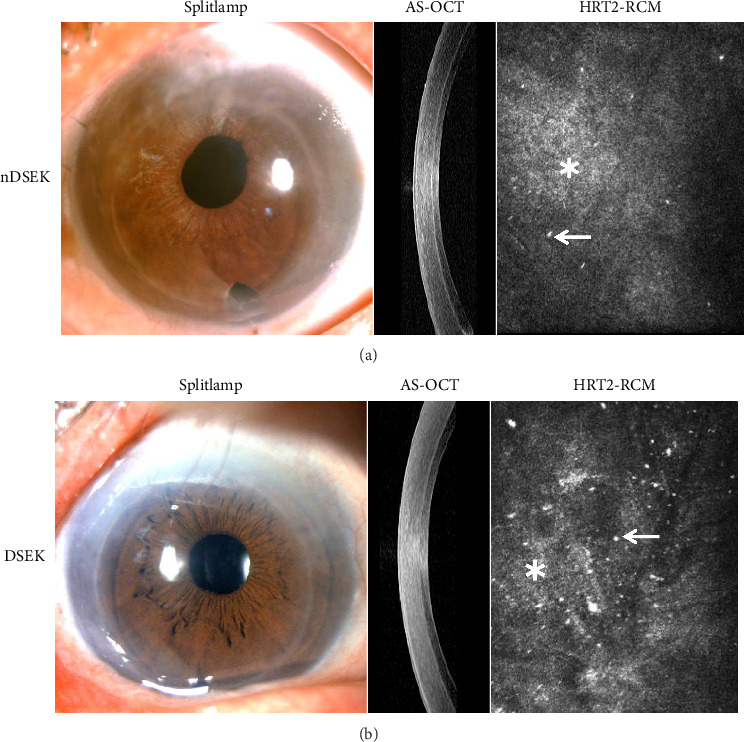
Representative photographs by slit-lamp microscopy, AS-OCT, and in vivo laser confocal microscopy examination. At 12 months postoperatively, all cases exhibited corneal transparency with secure adhesion of the endothelial graft to the recipient bed. Under slit-lamp microscopy, no clinically visible differences were observed between the nDSEK (a) and DSEK (b) groups regarding corneal transparency and the donor–recipient interface. In vivo laser confocal microscopy revealed particles (white arrow) of varying sizes and reflective intensities, as well as interface haze (white asterisk), at the donor–recipient interface in both the nDSEK (a) and DSEK (b) groups.

Further observation at the donor–recipient interface using in vivo laser confocal microscopy revealed that haze characteristically presents as a diffuse, poorly demarcated, highly reflective cloud-like pattern, while particles appear as discrete, variably sized, well circumscribed, highly reflective punctate structures. In addition, in vivo confocal microscopy identified particles of varying sizes and reflective intensities, along with differing degrees of haze, at the donor–recipient interface in both the nDSEK and DSEK groups (Figure 1).

In contrast to DSEK eyes, at 1 month postoperatively, in vivo confocal microscopy was able to detect enlarged, sparse, and indistinct outlines of recipient corneal endothelial cells at the donor–recipient interface in some nDSEK eyes (Supporting Figure S1). Representative photographs from slit-lamp microscopy, AS-OCT, and in vivo confocal microscopy are shown in Figure 1.

### 3.3. The Donor–Recipient Interface Particle Density and the Mean Pixel Intensity Units of Interface Haze Was Measured Quantitatively Using ImageJ

At 12 months postoperatively, the mean interface particle density was 631.97 ± 143.95 particles/mm^2^ in the nDSEK group and 518.20 ± 121.72 particles/mm^2^ in the DSEK group. A few eyes in the nDSEK group exhibited more noticeable interface particles during slit-lamp examinations, predominantly showing pigmented particles. The difference in mean interface particle density between the nDSEK and DSEK groups was statistically significant (*p*=0.001). Regarding donor–recipient interface haze, the mean pixel intensity units were higher in the nDSEK group (78.16 ± 13.74) compared with the DSEK group (64.21 ± 14.78) at 12 months postoperatively, with statistically significant between the two groups (*p* < 0.001).

### 3.4. Visual Outcomes

At 12 months postoperatively, the mean UCVA (logMAR) improved from 1.92 ± 0.26 to 0.46 ± 0.12 in the nDSEK group and from 1.85 ± 0.24 to 0.40 ± 0.10 in the DSEK group. Similarly, the mean BCVA (logMAR) improved from 1.92 ± 0.26 to 0.37 ± 0.11 in the nDSEK group and from 1.85 ± 0.24 to 0.34 ± 0.10 in the DSEK group. However, there were no statistically significant differences between the two groups in the improvement of either UCVA (*p*=0.077) or BCVA (*p*=0.149) (Table S1).

### 3.5. Correlation Analysis

Further correlation analysis showed significant correlation between interface particle density and pixel intensity units of donor–recipient interface haze in either the nDSEK group (correlation coefficient: 0.716, *p* < 0.001) or the DSEK group (correlation coefficient:0.618, *p* < 0.001), but no significant correlation between interface particle density and postoperative BCVA in either the nDSEK group (correlation coefficient: −0.028, *p*=0.883) or the DSEK group (correlation coefficient: 0.111, *p*=0.525) nor between pixel intensity units of donor–recipient interface haze and postoperative BCVA in either group (nDSEK: correlation coefficient: −0.080, *p*=0.670 and DSEK: correlation coefficient: −0.210, *p*=0.227).

### 3.6. Graft Dislocation

Graft dislocation occurred in one eye (3.2%) of the nDSEK group and one eye (2.9%) of the DSEK group on the first postoperative day. In all cases, successful rebubbling was performed. There were no statistically significant differences in the rate of graft dislocation between the nDSEK and DSEK groups (*p*=0.931).

## 4. Discussion

Standard DSEK or DSAEK involves removing the recipient's Descemet membrane and endothelial cells complex, which is crucial to avoid potential postoperative issues like graft dislocation [1]. Conversely, a few studies have indicated that nDSEK, which skips this step in patients without Descemet membrane abnormalities, can lead to positive outcomes, especially in cases of failed penetrating keratoplasty (PK) and may prevent original PK wound dehiscence [2–5]. However, there is limited and inconsistent research comparing these procedures in terms of changes at the donor–recipient interface and their impact on graft dislocation and visual outcomes.

In our current study, all cases showed corneal clarity and stable adhesion of the endothelial graft to the recipient bed at 12 months postoperatively, with no clinically observable differences regarding corneal transparency and the donor–recipient interface between the nDSEK and standard DSEK groups as assessed by slit-lamp microscopy. To our knowledge, this is the first study comparing the in vivo confocal microscopy donor–recipient interface features between the nDSEK and standard DSEK eyes. In vivo confocal microscopy detected particles of varying sizes and reflective intensities, as well as haze of different intensities at the donor–recipient interface in both groups. The analysis revealed that nDSEK—where the removal of Descemet's membrane and endothelial cell complex was omitted—exhibited a higher density of interface particles and increased haze compared to standard DSEK. However, these changes did not negatively impact the improvement in postoperative vision. A study utilizing a rabbit model similarly noted greater interface haze in the nDSAEK group compared with the standard DSAEK group, although this difference was not statistically significant [8]. Regarding postoperative BCVA improvement, our findings indicated that the nDSEK group achieved a visual acuity enhancement comparable with that of the standard DSEK group. These results suggest that while preserving the recipient's Descemet membrane and endothelial cells complex in nDSEK may lead to an increase in interface particle density and haze, it does not compromise visual outcomes when compared with standard DSEK.

Further correlation analysis revealed a significant relationship between interface particle density and pixel intensity units of donor–recipient interface haze in both the nDSEK and DSEK groups. However, no correlation was identified between interface particle density and postoperative BCVA in either group. Similarly, no correlation was observed between the pixel intensity of donor–recipient interface haze and postoperative BCVA in both the nDSEK and DSEK groups. These findings suggest that neither particle density nor haze at the interfaces had a significant impact on postoperative visual acuity outcomes in either group.

Some previous clinical studies on DSAEK reported similar outcomes but did not compare various endothelial grafting techniques, unlike our study, which contrasted the nDSEK and DSEK groups. Kobayashi et al. [9] observed that interface particle density decreased over 6 months postsurgery in DSAEK, suggesting that it may not directly correlate with postoperative BCVA. Furthermore, two studies—one conducted by Ferrari et al. in DSAEK [10] and the other by Espana et al. in DSEK [11]—also found no significant relationship between interface particle density and visual acuity, which aligns with our results. Interestingly, previous studies [9, 11] indicated that these donor–recipient interface particles tend to diminish over time, similar to lamellar particles observed after laser in situ keratomileusis. These particles are believed to have varying origins; temporary particles may consist of cellular debris or inflammatory cells, while more permanent particles could be metallic in composition [12].

A study by Kobayashi et al. [9] also found that interface haze decreased within 6 months postsurgery in DSAEK. However, the small sample size and short follow-up period in their study left it unclear whether there was a direct relationship between the observed interface haze and postoperative BCVA. Consistent with our findings, Espana and Huang [11] reported no significant correlation between interface haze and postoperative BCVA in DSEK. In contrast, Ferrari's study [10] identified a significant correlation between interface haze and postoperative BCVA in DSAEK.

Our study found no statistically significant difference in the rate of graft dislocation between the nDSEK group (3.2%) and the DSEK group (2.9%), both of which were lower than the average reported dislocation rate of 14.5% [13]. These results indicate that preserving the recipient's Descemet membrane and endothelial cell complex does not increase the risk of graft dislocation. This finding aligns with the observations of Caldwell et al. [2] and challenges the conventional wisdom surrounding standard DSEK or DSAEK procedures, which traditionally assert that the removal of the recipient's Descemet membrane and endothelial cell complex is necessary. It has been believed that the presence of these structures at the donor–recipient interface could impede effective graft attachment, potentially leading to dislocation [1].

Limited studies had investigated the postoperative fate of recipient's Descemet membrane and endothelial cells complex at the donor–recipient interface. An animal study revealed that 2 weeks post-nDSAEK, the preserved recipient Descemet membrane and endothelial cells complex were still present at the interface, with the endothelial cells expressing Na+/K + -ATPase protein, indicating that these cells could remain viable for at least 2 weeks [14]. Similarly, another study demonstrated that in an ex vivo culture model of human cornea, healthy endothelial-Descemet membrane grafts inserted into a stromal pocket could survive for up to 3 weeks, with a cell mortality rate of 30% [15]. In a rabbit model [8], all eyes treated with nDSAEK exhibited a time-dependent decline in the density of recipient's endothelial cells over a 3-month period. By the third postoperative month, cell density had reduced to 60%, and the cells no longer stained positive for Na+/K + -ATPase, suggesting a gradual loss of physiological function through slow apoptosis. In our current study, at 1 month postoperatively, in vivo confocal microscopy could detect enlarged, sparse, and indistinct outlines of recipient corneal endothelial cells at the donor–recipient interface in nDSEK eyes, although it could not confirm the viability of these cells. By 3 months after surgery, the corneal endothelial cells were no longer detectable. However, throughout the study, AS-OCT consistently revealed a persistent homogeneous band with moderate to low reflectivity at the donor–recipient interface in nDSEK eyes, which was presumed to represent the recipient's Descemet membrane. Furthermore, a clinical study [16] reported that histological and ultrastructural examinations conducted 1 year after 1 year after nDSEK confirmed the absence of endothelium at the donor–recipient interface, while the recipient's Descemet membrane remained intact, exhibiting a normal homogeneous structure without fibrous scar formation on either side.

A retrospective study by Omoto et al. compared nDSAEK and DSAEK in patients with non-Fuchs-type bullous keratopathy, finding comparable 5-year outcomes in postoperative visual acuity, graft survival rates, and complications [17]. Our results align with theirs, indicating similar effectiveness between nDSEK and DSEK. Although our preliminary study found that omitting this step did not hinder visual improvement or effective graft attachment in the treatment of corneal endothelial decompensation, it is important to note that in vivo laser confocal microscopy results showed that nDSEK exhibited more interface particles and haze compared with standard DSEK. Haze is known to impact contrast sensitivity, which may affect visual function. Therefore, our preliminary findings do not to alter established surgical practices (standard DSEK, DSAEK, and DMEK all involve stripping and removing the recipient's Descemet membrane and endothelial cell complex). In general, we still recommend the removal of the Descemet membrane and endothelial cell complex during surgery, particularly in cases with significant Descemet membrane abnormalities and retrocorneal fibrous membranes, who are not suitable candidates for nDSEK and should undergo Descemet membrane stripping. For instance, in cases of Fuchs endothelial dystrophy, nDSEK is not recommended due to the potential impact of pathological guttata on visual improvement [18]. In addition, patients with iris–corneal endothelial syndrome and those with retrocorneal fibrous membranes due to severe inflammatory responses also require complete Descemet membrane stripping.

For less experienced surgeons, nDSEK may offer advantages in certain cases. For example, in patients with a history of penetrating keratoplasty, eliminating the Descemet membrane stripping step can help avoid potential wound dehiscence associated with conventional Descemet membrane stripping [19]. In cases of congenital hereditary endothelial dystrophy, when Descemet membrane stripping is challenging and visibility is poor [20], overly pursuing complete Descemet membrane stripping may inadvertently damage the posterior corneal stroma.

This study has several limitations. First, as a retrospective study, it inherently carries certain weaknesses. Although image analysis was performed under blinded conditions, selection bias may have been introduced due to reliance on existing clinical and imaging data. Furthermore, the heterogeneity in image quality could potentially affect the robustness of our analytical outcomes. The small sample size and uneven group distribution also limit the strength and generalizability of our conclusions. In addition, the follow-up duration was relatively short. While haze is known to impact contrast sensitivity, we did not assess contrast sensitivity in this study, which restricts a comprehensive evaluation of visual function between nDSEK and DSEK. To validate these findings, a well-designed randomized controlled trial with a larger sample size and longer follow-up period is warranted in the future.

## 5. Conclusions

nDSEK simplified the procedure by eliminating the need to remove the recipient's Descemet membrane and endothelial cells complex. In vivo laser confocal microscopy showed that nDSEK exhibited more interface particles and haze than standard DSEK. However, the omission of this step in nDSEK did not hinder the improvement of visual outcomes or effective graft attachment in the treatment of corneal endothelial decompensation, compared with standard DSEK, provided there were no significant abnormalities in the Descemet membrane or retrocorneal fibrous membrane.

## Figures and Tables

**Table 1:** Demographic characteristics and baseline clinical data of patients.

	nDSEK (*n* = 31)	DSEK (*n* = 35)	*p* value
Age (years)	61.97 ± 17.15	61.51 ± 13.59	0.905
Female	11 (35.5%)	19 (54.3%)	0.126
Donor age (years)	45.61 ± 11.91	46.51 ± 9.55	0.735
Donor ECD (cells/mm^2^)	2816.77 ± 142.59	2861.06 ± 143.70	0.214
Preoperative BCVA (LogMAR)	1.92 ± 0.26	1.85 ± 0.24	0.218
Preoperative lens status			0.591
Pseudophakic	20 (64.5%)	25 (77.8%)	
Aphakic	4 (12.9%)	2 (5.7%)	
Phakic	7 (22.6%)	8 (22.9%)	

*Note:* age, donor age, donor ECD, and BCVA are expressed as the mean ± standard deviation and gender and lens status are presented as counts and percentages.

Abbreviations: BCVA, best-corrected visual acuity; DSEK, Descemet stripping endothelial keratoplasty; ECD, endothelial cell density; LogMAR, logarithm of minimal angle of resolution; nDSEK, non-Descemet stripping endothelial keratoplasty.

## Data Availability

The data that support the findings of this study are available on request from the corresponding author. The data are not publicly available due to privacy or ethical restrictions.

## Supporting Information

Supporting Information 1 Supporting 1: Figure S1: In vivo laser confocal microscopy examination of the recipient corneal endothelial cells at the donor–recipient interface. In contrast to DSEK eyes, at 1 month postoperatively, in vivo confocal microscopy was able to detect enlarged, sparse and indistinct outlines of recipient corneal endothelial cells (white arrow) at the donor–recipient interface in some nDSEK eyes.

Supporting Information 2 Supporting 2: Figure S2: Representative photographs from the AS-OCT assessment. At 12 months postoperatively, a homogeneous band of moderate to low reflectivity was observed at the recipient (white asterisk)—donor (white arrowhead) interface in the (A) nDSEK eyes, which is presumed to represent the recipient's Descemet membrane (white arrow). In contrast, no similar band was detected in the (B) DSEK eyes.

Supporting Information 3 Supporting 3: Table S1. Visual outcomes in the nDSEK and DSEK before and at potoperative month 12, the visual outcomes in UCVA and BCVA over time in the two groups were compared using a repeated measures analysis of variance.
