# Supplementary material for: Comparison and Correlation of the Donor–Recipient Interface Changes and Visual Outcomes Between nDSEK and DSEK
Source: J Ophthalmol. 2025 Mar 12;2025:2066562. doi: 10.1155/joph/2066562 (PMC11991815; doi:10.1155/joph/2066562)
Supplement: Supporting Information 1 — Supporting 1: Figure S1: In vivo laser confocal microscopy examination of the recipient corneal endothelial cells at the donor–recipient interface. In contrast to DSEK eyes, at 1 month postoperatively, in vivo confocal microscopy was able to detect enlarged, sparse and indistinct outlines of recipient corneal endothelial cells (white arrow) at the donor–recipient interface in some nDSEK eyes. [file 2066562.f1.pptx]

## Slide 1
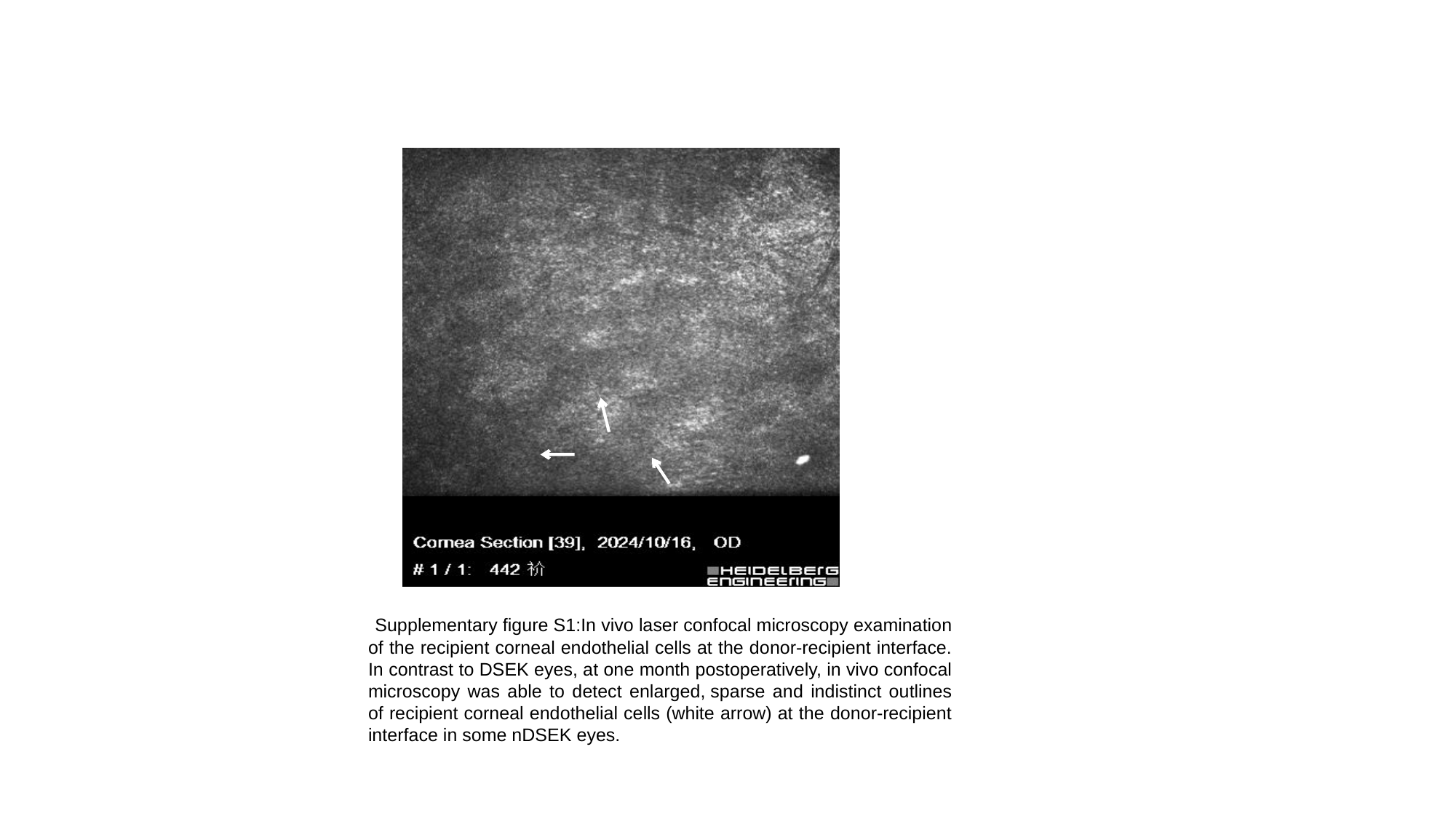

Supplementary figure S1:In vivo laser confocal microscopy examination of the recipient corneal endothelial cells at the donor-recipient interface. In contrast to DSEK eyes, at one month postoperatively, in vivo confocal microscopy was able to detect enlarged, sparse and indistinct outlines of recipient corneal endothelial cells (white arrow) at the donor-recipient interface in some nDSEK eyes.
