# Supplementary material for: Comparison and Correlation of the Donor–Recipient Interface Changes and Visual Outcomes Between nDSEK and DSEK
Source: J Ophthalmol. 2025 Mar 12;2025:2066562. doi: 10.1155/joph/2066562 (PMC11991815; doi:10.1155/joph/2066562)
Supplement: Supporting Information 2 — Supporting 2: Figure S2: Representative photographs from the AS-OCT assessment. At 12 months postoperatively, a homogeneous band of moderate to low reflectivity was observed at the recipient (white asterisk)—donor (white arrowhead) interface in the (A) nDSEK eyes, which is presumed to represent the recipient's Descemet membrane (white arrow). In contrast, no similar band was detected in the (B) DSEK eyes. [file 2066562.f2.pptx]

## Slide 1
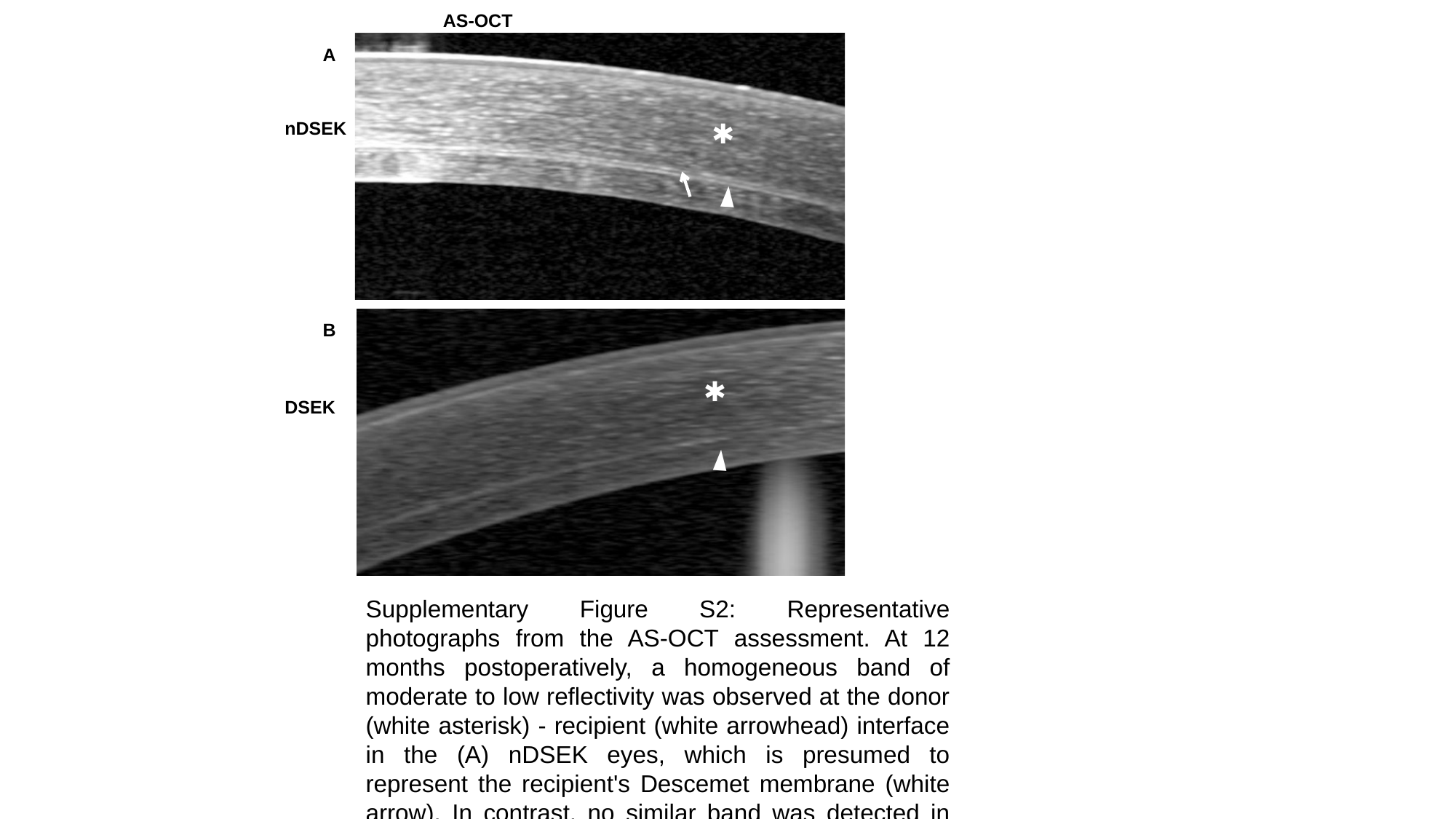

AS-OCT
 nDSEK
✱
►
✱
 DSEK
►
A
B
Supplementary Figure S2: Representative photographs from the AS-OCT assessment. At 12 months postoperatively, a homogeneous band of moderate to low reflectivity was observed at the donor (white asterisk) - recipient (white arrowhead) interface in the (A) nDSEK eyes, which is presumed to represent the recipient's Descemet membrane (white arrow). In contrast, no similar band was detected in the (B) DSEK eyes.
