# Supplementary material for: Comparison and Correlation of the Donor–Recipient Interface Changes and Visual Outcomes Between nDSEK and DSEK
Source: J Ophthalmol. 2025 Mar 12;2025:2066562. doi: 10.1155/joph/2066562 (PMC11991815; doi:10.1155/joph/2066562)
Supplement: Supporting Information 3 — Supporting 3: Table S1. Visual outcomes in the nDSEK and DSEK before and at potoperative month 12, the visual outcomes in UCVA and BCVA over time in the two groups were compared using a repeated measures analysis of variance. [file 2066562.f3.docx]

| Table S1. Visual outcomes in the nDSEK and DSEK before and at potoperative month 12 | | | |
| --- | --- | --- | --- |
|  | nDSEK (n=31) | DSEK(n=35) | P value |
| Improvement in UCVA |  |  | 0.077 |
| Preoperative UCVA (LogMAR) | 1.92±.26 | 1.85±0.24 |  |
| UCVA at 12 months (logMAR) | 0 .46±0 .12 | 0.40±0.10 |  |
| Improvement in BCVA |  |  | 0.149 |
| Preoperative BCVA (LogMAR) | 1.92±.26 | 1.85±0.24 |  |
| BCVA at 12 months (LogMAR) | 0.37 ± 0.11 | 0.34 ± 0.10 |  |

nDSEK: non-Descemet stripping endothelial keratoplasty; DSEK: Descemet stripping endothelial keratoplasty; UCVA: uncorrected visual acuity; BCVA: best corrected visual acuity; LogMAR: logarithm of minimal angle of resolution; UCVA and BCVA were expressed as mean ± standard deviation; Preoperative UCVA was equal to preoperative BCVA (no improvement with correction).The visual outcomes in UCVA and BCVA over time in the two groups were compared using a repeated measures analysis of variance.
